# Rachianesthésie pour césarienne: facteurs de risque d'apparition de scores d'Apgar inférieur à 7 chez les nouveau-nés malgaches

**DOI:** 10.11604/pamj.2014.19.193.3392

**Published:** 2014-10-23

**Authors:** Tsiorintsoa Yvonne Rasolonjatovo, Bako Minosoa Gilberthe Ravololonirina, Zely Arivelo Randriamanantany, Nasolotsiry Enintsoa Raveloson

**Affiliations:** 1 Hôpital Universitaire de Gynécologie et Obstétrique de Befelatanana, Antananarivo, Madagascar; 2 Centre Hospitalier Universitaire de Fianarantsoa, Madagascar; 3 Centre Hospitalier Universitaire Joseph Raseta de Befelatanana, Antananarivo, Madagascar

**Keywords:** Rachianesthésie, score d′Apgar, éphédrine, hypotension artérielle, Spinal anesthesia, Apgar score, ephedrine, hypotension

## Abstract

**Introduction:**

La rachianesthésie est courante en obstétrique. L'hypotension artérielle maternelle apparaît dans 50-80%. Elle affecte l’équilibre acido-basique et l’état clinique du nouveau-né. Pour y remédier, l'usage de vasoconstricteurs est incontournable. L’éphédrine était de loin de premier choix en obstétrique. Dans les pays développés, elle est co-administrée avec la phényléphrine. A Madagascar, seule l’éphédrine reste disponible. Notre étude consiste à déterminer les facteurs de risque d'apparition du score d'Apgar inférieur à 7 chez les nouveau-nés.

**Méthodes:**

Une étude rétrospective transversale, analytique, était effectuée à la Maternité de Befelatanana, Antananarivo Madagascar (Décembre 2010-Décembre 2011). Nous avons inclus 344 césariennes opérées sous rachianesthésie. Le critère principal de jugement était l'observation de score d'Apgar inférieur ou égal à 7 à la première minute. Les données étaient analysées sur logiciel Epi Info version 6.04 (IC à 95%, p bilatéral < 0.05).

**Résultats:**

Le score d'Apgar était inférieur ou égal à 7 dans 42%. Les facteurs de risque retrouvés étaient la césarienne en urgence, la dose d’éphédrine dépassant 30mg ainsi que la baisse de la pression artérielle diastolique supérieure à 10% par rapport à sa valeur initiale (p respectif <<0.05).

**Conclusion:**

La précarité de l’état clinique des nouveau-nés à la naissance est multifactorielle. La baisse de la pression artérielle diastolique associée à une dose élevée d’éphédrine est néfaste. De plus, l'augmentation de la catécholaminémie maternelle puis fœtale l'aggrave.

## Introduction

L'hypotension artérielle maternelle post rachianesthésie (RA) apparaît dans 50-80% [1]. Elle affecte l’équilibre acido-basique du fœtus et son état clinique [2]. Pour y remédier, nombreuses mesures préventives et curatives étaient élaborées [3, 4]. L'usage de vasoconstricteurs est indispensable [5, 6]. L’éphédrine était le vasoconstricteur de premier choix en obstétrique [7, 8]. Actuellement dans les pays développés, l’éphédrine se co-administre avec la phényléphrine [1, 7–9]. Cette association procure de meilleurs effets hémodynamiques maternels. L’équilibre acido-basique fœtal est également amélioré [5, 6]. A Madagascar, la phényléphrine n'est pas encore disponible. En 2009, la césarienne représentait 14% des accouchements effectués à la Maternité de Befelatanana, Centre Hospitalier Universitaire d'Antananarivo Madagascar. La RA était effectuée dans 8% des cas, dont 27% des naissances vivantes avaient un score d'Apgar < 7 à la première minute (Source Maternité Befelatanana). Depuis Janvier 2010, le taux de césarienne augmentait de 35%. Nous avons alors réalisé cette étude dans le but de déterminer les facteurs de risque pouvant contribuer à l'apparition du score d'Apgar ≤ 7 chez les nouveau-nés malgaches.

## Méthodes

Il s'agit d'une étude rétrospective transversale analytique. Elle était effectuée à la Maternité de Befelatanana. Les césariennes opérées sous RA allant du mois de Décembre 2010 au mois de Décembre 2011 étaient répertoriées. Les critères d'inclusion concernaient les RA ayant reçues un pré-remplissage vasculaire de 500ml de cristalloïde suivi d'injection intrathécale de bupivacaïne hyperbare 0,5% associée à 25µg de fentanyl (dose de la bupivacaïne adaptée à la taille de la patiente). Nous avons exclu, les césariennes indiquées pour hypertensions artérielles (avec ou sans prééclampsie) et les chorioamniotites. Le critère principal de jugement était l'observation de score d'Apgar ≤ 7 à la première minute (M1). Suivant ce critère principal, les données étaient classées en deux sous groupes. Le sous groupe des patientes ayant un nouveau-né à score d'Apgar ≤ 7 était noté Gmalade. Le sous groupe des patientes ayant un nouveau-né à score d'Apgar > 7 était noté Gtémoin. Les variables relevées étaient: démographique (âge, gestité, parité), cliniques (pression artérielle systolique ou PAS avant la RA, PAS la plus basse observée, pression artérielle diastolique ou PAD avant la RA, PAD la plus basse observée, score d'Apgar à M _1_ (SAM _1_), la quantité totale en millilitre de cristalloïde perfusée ainsi que dose totale d’éphédrine injectée jusqu'au clampage du cordon ombilical, la dose de bupivacaïne hyperbare injectée en intrathécale et la nature programmée ou non de la césarienne. Les données étaient informatisées sur logiciel Excel. L'analyse statistique était effectuée sur logiciel Epi Info version 6.04. L'ANOVA était réalisée pour comparer les caractéristiques de la population d’étude entre les sous groupes. Le Test de Chi ^2^ ainsi que le calcul du risque relatif (IC 95%) étaient effectués pour rechercher les facteurs de risque. Une différence est statistiquement significative pour une valeur de p bilatéral inférieur à 0.05.

## Résultats

Durant cette période, 344 césariennes opérées sous RA étaient inclues. Les césariennes étaient non programmées dans 65,5% des cas. Le SAM _1_ ≤7 représentait 42% des cas (145 cas sur 344). La comparaison des caractéristiques de la population d’étude entre les sous groupe Gmalade et Gtémoin est rapportée dans le Tableau 1. Les résultats de l'analyse des facteurs de risque d'apparition de SAM _1_ ≤ 7 sont relatés dans le Tableau 2.

**Tableau 1:** Comparaison entre les sous-groupes

Variables	Gmalade (n=145)	Gtémoin (n=199)	p
Age (ans)	29±7	28±7	0.33
Poids (kg)	59±3	58±4	0.32
Gestité	2±1	2±1	0.93
Parité	2±1	2±1	0.93
Cristalloïde (ml)	1262±491	1200±463	0.34
PAS initiale (mmHg)	128±16	128±19	0.54
PAS la plus basse (mmHg)	120±11	122±93	0.24
PAD initiale (mmHg)	79±13	78±14	0.47
PAD la plus basse (mmHg)	71±9	74±9	0.04
Ephédrine (mg)	79±14	63±22	0.01
Bupivacaïne (mg)	14±1	14±2	0.71

Résultats exprimés en moyenne (écart-type); ANOVA

**Tableau 2:** Analyse des facteurs de risque d'apparition de SAM_1_ ≤ 7

Variables	Gmalade (n=145)	Gtémoin (n=199)	RR (IC 95%)	p
**Baisse PAD**				
Baisse < 10%	54 (37)	139 (70)	2,15 (1,66–2,80)	< 0.000
Baisse ≥ 10%	91 (63)	60 (30)		
**Ephédrine**				
Ephédrine < 30mg	10 (7)	54 (27)	3,09 (1,72–5,52)	< 0.000
Ephédrine ≥ 30mg	125 (93)	145 (73)		
**Césariennes**				
En urgence	115 (79)	109 (55)	2,05 (1,47–2,87)	< 0.000
Programmée	30 (21)	90 (45)		

Résultats exprimés en effectif (pourcentage); RR: risque relatif; IC 95%: Intervalle de Confiance à 95%; Test de Chi^2^

## Discussion

Nos résultats ont fait ressortir trois facteurs de risque d'apparition de SAM _1_ ≤ 7. Ce sont: la césarienne en urgence (p9,12, 13]. Diverses mesures thérapeutiques, pharmacologiques et physiques, étaient mises au point dans le but d'atténuer l'hypotension artérielle maternelle post-RA. Ce sont le pré et co-remplissage vasculaire avec du cristalloïde, la mise en décubitus latéral gauche (DLG) de 10 à 15° et l'usage de vasoconstricteurs [4, 10, 14]. e remplissage vasculaire serait efficace pour une quantité totale de 20ml/kg. Dans notre série, les deux sous groupes étaient comparables (Tableau 1). La mise en léger DLG favorise le retour veineux [4, 10, 14]. Dans le cas contraire, on va observer une hypotension artérielle persistante malgré un remplissage vasculaire bien conduit [4]. De nombreuses définitions de l'hypotension artérielle maternelle post-RA étaient rapportées [15]. Aucun consensus n’était établi pour la définir [15, 16]. La PAS est la plus considérée notamment une baisse de plus de 20% de sa valeur de base [17]. Dans notre série, il était ressorti que seule la baisse de la PAD de plus de 10% de sa valeur initiale constituait un facteur de risque pour le nouveau-né (Tableau 1, Tableau 2). Le positionnement de la mère en léger DLG serait impliqué. Aucun de nos patientes n’était mis en léger DLG après la RA. Par conséquent, le poids de l'ensemble utérus-fœtus va gêner la circulation sanguine aorto-cave. Par conséquent, le pré charge va baisser. Ce qui aboutit à la baisse de la PAD [4]. LL'opération césarienne constitue un stress maternel [18]. Le stress favoriserait également l'augmentation de la catécholaminémie maternelle puis fœtale [19]. Son effet chez le fœtus est d'autant plus marqué lorsque la césarienne est réalisée en urgence [8]. Dans notre série, la césarienne en urgence concernaient 79% des nouveau-nés du sous groupe Gmalade contre 55% dans le sous groupe Gtémoin (Tableau 2). A cette élévation spontanée de la catécholaminémie maternelle s'ajoute l'effet des vasoconstricteurs. L'usage de vasoconstricteurs est incontournable après une RA [8, 9, 20]. L’éphédrine est une catécholamine de synthèse à action ß adrénergiques. Elle stimule la libération pré synaptique de noradrénaline et du métabolisme fœtal. Ce qui aboutit à un état d'acidose fœtal [8, 21]. De nombreuses études ont démontré que l'acidose est corrélée aux doses totales d’éphédrine, variant de 20 à 50mg [4, 10, 22]. Dans notre série, une dose totale de plus de 30mg d’éphédrine constitue un facteur de risque d'apparition de SAM _1_ ≤7 (Tableau 2). u terme de cette étude, il serait indispensable d'améliorer certains points de notre protocole de prise en charge des opérations césariennes sous RA. La prévention reste primordiale. Le suivi régulier des grossesses, l'observance thérapeutique et la détection à temps des grossesses à risque de césarienne contribueraient sans doute à la réduction du taux des césariennes en urgence. En pratique clinique, l'optimisation de la circulation aorto-cave par la surélévation de la fesse droite à l'aide d'un coussin constituerait un moyen physique moins couteux et accessible dans les blocs opératoires moyennement équipés. Le co-remplissage vasculaire permettrait d'anticiper le traitement de l'hypotension artérielle. L’éphédrine reste le vasoconstricteur de premier choix dans nos blocs opératoires. Sa dose totale ne devra pas excéder les 30 mg. Cependant, sa co-administration à d'autres vasoconstricteurs serait souhaitable. Depuis une décennie, de nombreuses études ont démontré les avantages de cette co-administration phényléphrine-éphédrine. L'hypotension artérielle maternelle est atténuée et rapidement stabilisée. La lactatémie et le pH artériels des nouveau-nés sont améliorés [7, 15, 23–26]. Sur le plan économique, la mise au point d'un nouveau protocole était démontrée bénéfique [23]. Le coût des vasoconstricteurs a permis d’économiser 3000 euros pour 1000 césariennes [23].

## Conclusion

Cette étude rétrospective avait pour objectif de déterminer les facteurs de risque d'apparition de SAM _1_ ≤7 chez les nouveau-nés issus de césarienne sous RA. Trois facteurs de risque étaient ressortis à savoir la césarienne en urgence, la baisse de la PAD ≥10% de sa valeur initiale et la dose totale d’éphédrine injectée dépassant les 30mg. La stratégie d'amélioration de ces résultats reste multidisciplinaire. Une collaboration étroite et consensuelle entre anesthésistes, obstétriciens et pédiatres est indispensable.
